# Complete genome sequence of the calcite-precipitating bacterium *Cytobacillus kochii* strain ITBMC_116, isolated from a limestone karst in Vietnam

**DOI:** 10.1128/mra.01367-25

**Published:** 2026-02-11

**Authors:** Thiet M. Vu, Loan Q. Le, Thanh M. Luc, Khoa H. D. Do, Danh H. Nguyen, Kien T. Tran, Nhung T. T. Vu, Dung H. Nguyen

**Affiliations:** 1 Functional Genomics Research Center, NTT Hi-tech Institute, Nguyen Tat Thanh University 646116, Ho Chi Minh City, Vietnam; 2 Institute of Life Sciences, Vietnam Academy of Science and Technology 562485 , Ho Chi Minh City, Vietnam; 3 Biotechnology Department, Graduate University of Science and Technology, Vietnam Academy of Science and Technology 61797, Hanoi City, Vietnam; Rochester Institute of Technology, Rochester, New York, USA

**Keywords:** *Cytobacillus kochii*, microbially induced calcite precipitation, whole genome sequencing

## Abstract

We report the complete genome of calcite-precipitating *Cytobacillus kochii* strain ITBMC_116, isolated from limestone karst in Vietnam. Hybrid Illumina–Nanopore sequencing yielded a 4.83-Mbp chromosome and an 87.4-kbp plasmid. This resource supports studies of microbially induced calcite precipitation and biomineralization-based biotechnologies.

## ANNOUNCEMENT

Bacteria within the genus *Cytobacillus* are of increasing biotechnological interest due to their potential for microbially induced calcite precipitation (1). This biomineralization capability is critical for sustainable civil engineering applications, such as biocementation, soil stabilization, and the development of self-healing concrete (2). The isolation of novel, robust calcite-precipitating bacteria from unique environments provides valuable genetic resources for understanding and harnessing these capabilities. Here, we present the complete genome sequence of *Cytobacillus kochii* strain ITBMC_116, isolated from a limestone sample collected in the karst region of Ho Chi Minh City, Vietnam (geographical coordinates: 10.544574, 107.166390). The strain was deposited at the Department of Applied Microbiology, Institute of Life Sciences (contact: Dung H. Nguyen, email: nhdung@ils.vast.vn).

A single colony of strain ITBMC_116 was inoculated into 2 mL of Nutrient Broth medium (Oxoid) and cultured overnight (14 h) at 30°C with shaking. Total genomic DNA was extracted from the cell pellet using a DNA Miniprep Kit (BioBasic) and size-selected using magnetic beads (Beckman Coulter). The quality and quantity of the DNA sample were checked using agarose gel (1%) electrophoresis and Qubit 4 fluorometer (Thermo Fisher Scientific), respectively. Short-read sequencing was performed by KTest Science Co. Ltd. (Ho Chi Minh City, Vietnam) on an Illumina MiSeq platform (2 × 150 bp paired-end), generating 44,772,776 reads (~6.7 Gbp of data). Long-read sequencing was conducted in-house using an Oxford Nanopore Technologies (ONT) MinION Mk1B device with Ligation Sequencing Kit (SQK-LSK109) and an R9.4 flow cell, yielding 163,079 reads (~1.87 Gbp of data). For quality control, raw Illumina reads were assessed with FastQC (v0.11.9) and trimmed using fastp (3). ONT reads were base-called with Guppy (v6.5.7) in high-accuracy mode and filtered using NanoPlot (4) to retain reads ≥ 1,000 bp.

A hybrid assembly of the high-quality Illumina (44,480,667 reads) and ONT (103,313 reads) data was performed using Unicycler (v0.5.0) (5) and resolved two complete circular replicons, including a single chromosome (4,830,791 bp) with an average coverage depth of 1,345× and a GC content of 36.8% and plasmid pITBMC_116 (87,365 bp) with an average coverage depth of 1,941× and a GC content of 33%. The assembly quality was assessed using CheckM2 (v1.1.0) (6) for contamination (0.21%) and BUSCO (v5.8.0) (7) for completeness (96.9%).

Taxonomic identification using the Type Strain Genome Server (8) and GTDB-Tk (v2.3.2) (9) classified the isolate as *Cytobacillus kochii*, with *C. kochii* DSM 23667^T^ as the closest reference. Annotation via the NCBI Prokaryotic Genome Annotation Pipeline (PGAP, release 6.1) (10) predicted 5,015 chromosomal genes, including 4,802 protein-coding sequences (CDSs), 107 tRNAs, and 30 rRNAs. The plasmid carried 98 predicted CDS. Default parameters were used for all software.

## Data Availability

This Whole Genome Shotgun project has been deposited at GenBank under the BioProject accession number PRJNA1310224 and BioSample SAMN50770592. The complete chromosome and plasmid sequences are available under accession numbers JBQSLM010000001 and JBQSLM010000002, respectively. The chromsome sequence described in this paper is GenBank accession version CM126253.1. The raw Illumina and ONT reads have been deposited in the Sequence Read Archive (SRA) under accession numbers SRR36067571 and SRR36067570, respectively.
